# A DNA Vaccine Encoding Multiple HIV CD4 Epitopes Elicits Vigorous Polyfunctional, Long-Lived CD4^+^ and CD8^+^ T Cell Responses

**DOI:** 10.1371/journal.pone.0016921

**Published:** 2011-02-11

**Authors:** Daniela Santoro Rosa, Susan Pereira Ribeiro, Rafael Ribeiro Almeida, Eliane Conti Mairena, Edilberto Postól, Jorge Kalil, Edecio Cunha-Neto

**Affiliations:** 1 Laboratory of Clinical Immunology and Allergy-LIM60, Division of Clinical Immunology and Allergy, Department of Medicine, University of São Paulo School of Medicine, São Paulo, Brazil; 2 Heart Institute (InCor), University of São Paulo School of Medicine, São Paulo, Brazil; 3 Institute for Investigation in Immunology-INCT, São Paulo, Brazil; 4 Division of Immunology-Federal University of São Paulo-UNIFESP, São Paulo, Brazil; University of California San Francisco, United States of America

## Abstract

T-cell based vaccines against HIV have the goal of limiting both transmission and disease progression by inducing broad and functionally relevant T cell responses. Moreover, polyfunctional and long-lived specific memory T cells have been associated to vaccine-induced protection. CD4^+^ T cells are important for the generation and maintenance of functional CD8^+^ cytotoxic T cells. We have recently developed a DNA vaccine encoding 18 conserved multiple HLA-DR-binding HIV-1 CD4 epitopes (HIVBr18), capable of eliciting broad CD4^+^ T cell responses in multiple HLA class II transgenic mice. Here, we evaluated the breadth and functional profile of HIVBr18-induced immune responses in BALB/c mice. Immunized mice displayed high-magnitude, broad CD4^+^/CD8^+^ T cell responses, and 8/18 vaccine-encoded peptides were recognized. In addition, HIVBr18 immunization was able to induce polyfunctional CD4^+^ and CD8^+^ T cells that proliferate and produce any two cytokines (IFNγ/TNFα, IFNγ/IL-2 or TNFα/IL-2) simultaneously in response to HIV-1 peptides. For CD4^+^ T cells exclusively, we also detected cells that proliferate and produce all three tested cytokines simultaneously (IFNγ/TNFα/IL-2). The vaccine also generated long-lived central and effector memory CD4^+^ T cells, a desirable feature for T-cell based vaccines. By virtue of inducing broad, polyfunctional and long-lived T cell responses against conserved CD4^+^ T cell epitopes, combined administration of this vaccine concept may provide sustained help for CD8^+^ T cells and antibody responses- elicited by other HIV immunogens.

## Introduction

Despite the success of antiretroviral treatment, a safe and effective HIV vaccine is the most promising strategy for controlling the AIDS pandemic, especially in developing countries. HIV vaccine strategies that focus on the generation of virus-specific T-cell responses have the goal of limiting both transmission and disease progression by controlling HIV viral loads [1].

To date, two efficacy trials assessed HIV-specific cellular mediated immunity. The STEP vaccine trial developed by Merck used a replication-defective Ad5 vector, expressing Gag, Pol and Nef proteins from HIV-1 [2]. The results from this trial demonstrated that it neither prevented HIV-1 infection nor reduced viral load in subsequently infected subjects [3], [4]. Immunological analyses revealed that each vaccinated individual recognized an average of only three epitopes [5]. The narrowness of the induced immune responses may have been an important factor in the lack of vaccine efficacy [3]. Indeed, non-human primate studies have shown that vaccines that induced broad CD8^+^ and CD4^+^ T cell responses can control peak SIV viremia [6]. In the recently reported RV144 HIV-1 vaccine trial conducted in Thailand, an immunization strategy based on recombinant canarypox priming followed by a protein boosting generated modest protection against the acquisition of HIV infection. Immunological analysis of samples from the study showed that the vaccine-induced immune response was essentially composed of CD4^+^ T cells and binding antibodies; no IFNγ or IL-2 secreting HIV-specific CD8+ T cells were detected [7]. However, the same immunogens induced cytotoxic immune responses in a minority of vaccinees in previous studies [8]. At any event, data from the RV 144 trial supported the notion that CD4+ T cells could play a protective role in HIV vaccine-induced immunity.

CD4+ T cells can contribute to protection against viral infection by both indirect and direct manners [9]–[11]. CD4^+^ T cell can help induce and maintain CD8^+^ and B cell responses. The main contribution of CD4^+^ T cells is to provide help to full differentiation and maintenance of cytotoxic CD8^+^ T cells and B cells. They promote the generation of CD8^+^ cytotoxic T cell response (CTL) able to control viral replication [12]–[14] as well as mobilization of CTLs to peripheral sites of infection [15]. Furthermore, CD4^+^ T cells can promote B cell differentiation into plasma cells to produce neutralizing antibodies and assist memory B cells responses to re-infection [16]. CD4^+^ T cells can also exert direct and indirect antiviral effects in retroviral infection. The outcome of retroviral infection depends on the magnitude and duration of virus-specific CD4^+^T cell responses [17]. A direct antiviral effect of CD4+ T cells was also observed in SIV infection. CD4^+^ T cells induce apoptosis of SIV-infected macrophages [18]. The presence of SIV-specific CD4^+^ T cell responses with a cytotoxic phenotype was associated with the control of rebounding viremia in CD8^+^ depleted SIV-infected macaques [19]. Further in support of a protective role for CD4^+^ T cell responses, it has been shown that elite controller SIV-infected macaques mount broad CD4^+^-specific T cell responses, and that certain macaque class II alleles are associated with significantly decreased viral loads [20].

Vaccination strategies that induced broad, polyfunctional and long-lasting SIV-specific CD4^+^ and CD8^+^ T cell responses were able to lower viral load after repeated mucosal challenge in the absence of antibodies [6], [21]. Recently, the impact of CD4^+^ T cell help on the generation of adaptive CD8^+^ T cell responses in SIV infection of nonhuman primates was evaluated. Indeed, vaccination in the absence of CD4^+^ T cells reduced protection mediated by CD8^+^ T cells after SIV infection [22]. Moreover, passive immunization with a SIV-specific neutralizing antibody led to a significant increase in polyfunctional SIV-Gag specific CD4^+^ T cells, and the frequency of these cells was inversely correlated with the plasma viral load during chronic infection [23]. Although a major concern is that CD4^+^ T cells induced by vaccination can serve as immediate HIV-1 targets, to date no evidence exists that CD4^+^ T cell activation or vaccine-induced CD4^+^ T cells results in heightened HIV-1 acquisition or viremia after infection [5], [7]. Indeed, in SIV-challenged rhesus macaques, data suggest that CD8+ T cell function has a greater impact on viremia than the activation status of CD4^+^ target cells [24]. In addition, Ad5-based SIV vaccine regimens induce powerful CD4+ T cell responses in animals that control viremia [6]. It could thus be hypothesized that a vaccine inducing potent CD4^+^ T cell responses might have a protective effect in HIV/SIV infection, possibly due to cognate help, leading to induction, maintenance and differentiation of CD8^+^ cytotoxic and/or B cell responses. It is thus clear that a successful HIV vaccine should also induce a strong CD4^+^ T cell response [25]. Our group has recently identified conserved CD4 epitopes from HIV that were able to bind to multiple HLA-DR molecules. Peptides encoding such epitopes were recognized by PBMC from over 90% of HIV-1 infected individuals [26]. We recently reported that a DNA vaccine encoding such promiscuous epitopes (HIVBr18) was able to induce broad specific CD4^+^ and CD8^+^ T cell responses in mice transgenic to common HLA class II alleles (HLA-DR2, -DR4, -DQ6, -DQ8) [27]. Significantly, 16 out of the 18 encoded epitopes were recognized. However, the functional profile induced by the vaccine was not evaluated in the HLA class II transgenic mice.

Several lines of evidence suggest an important role for specific polyfunctional T cell responses in immune control of different pathogens [28]. In HIV infection, the presence of polyfunctional T cell responses has been associated with both the control of virus replication [29] and protection from disease progression [14], [30]–[32]. Moreover, polyfunctional HIV-specific CD4^+^ T cells were found in the rectal mucosa of infected individuals that spontaneously control HIV replication, named elite controllers. The proportion of such cells directly correlated with the total magnitude of the mucosal specific CD8^+^ T cell responses [14]. The maturation status of T cells is also an important issue. Central memory T cells are thought to ensure the long-term maintenance of antiviral responses due to their long half-life and self-renewal capacity [33]. HIV controllers have a preserved central memory CD4^+^ T cell compartment and sustain an effector memory CD4^+^ T cell population [34]. A vaccine able to induce SIV-specific effector memory CD4^+^ and CD8^+^ T cell responses, in the absence of neutralizing antibodies, was able to prevent establishment of progressive systemic infection after mucosal challenge with a highly pathogenic SIV [21]. Indeed, memory T cells have been associated with long-term vaccine induced protection [35].

In the current study, we have evaluated the polyfunctionality, longevity and memory phenotype of the HIV- specific T cell responses induced by HIVBr18, a DNA vaccine encoding promiscuous CD4 epitopes, in BALB/c mice. We found that HIVBr18 was able to induce high magnitude, broad and polyfunctional CD4^+^/CD8^+^ T cell responses, and 8/18 vaccine-encoded peptides were recognized. Moreover, the vaccine also generated long-lived central and effector memory CD4^+^ T cells, a desirable feature for T cell-based vaccines.

## Results

### Broad specific T cell responses following immunization with a DNA vaccine encoding promiscuous HIV-1 epitopes

To analyze whether the HIVBr18 vaccine could be immunogenic in BALB/c mice, we first performed an *in silico* analysis. For this purpose, we evaluated the ability of the HIV peptides to bind to BALB/c MHC molecules, using the PRED^BALB/c^ algorithm, a prediction algorithm specific for the H-2 Dd, Kd, I-Ad, and I-Ed molecules [30]. All HIV-1 peptides encoded by the vaccine were predicted to bind to BALB/c H-2^d^ class II molecules; most peptides were predicted to bind to at least one H-2^d^ class I molecule as well (Table S1).

**Figure 1:**
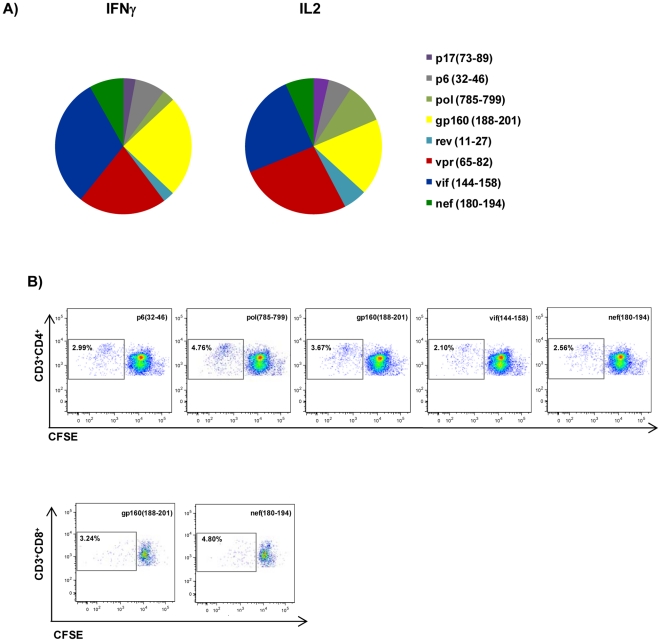
Immunization with HIVBr18 induces IFNγ and IL-2 secretion and proliferation against multiple HIV-1 epitopes. Two weeks after the last immunization with HIVBr18 or the empty pVAX1 vector, pooled spleen cells from 6 BALB/c mice were cultured in the presence of HIV-1 peptides (5 µM) or medium only. (A) Frequencies of HIV peptide-specific IFNγ (left pie chart) and IL-2 (right pie chart) secreting cells were measured by ELISPOT assay. The responses are shown by displaying each the number of SFU/10^6^ cells for each positive peptide as a proportion of the sum of SFU/10^6^ cells for all positive peptides. (B) Proliferative T cell responses were assessed by CFSE dilution assay. Splenocytes were labeled with CFSE (1.25 µM) and cultured for 5 days. After staining with fluorochrome-labeled anti-CD3, -CD4 and -CD8 monoclonal antibodies, cells were analyzed by flow cytometry. CFSE dilution on gated CD3^+^CD4^+^ or CD3^+^CD8^+^ cells was used as a readout for antigen-specific proliferation. Representative dot plots of CD4^+^ (upper panels) and CD8^+^ (lower panels) T cell proliferation (values in boxes represent % CFSE^low^ cells) of splenocytes from HIVBr18 immunized mice; Data are representative of nine independent immunization experiments.

To analyze whether immunization with HIVBr18 could induce specific T cell immune responses, BALB/c mice were immunized with HIVBr18 or the empty vector pVAX1. Fifteen days after the last dose, splenocytes from immunized mice were incubated with each of the 18 HIV-1 peptides encoded by the DNA vaccine, and specific IFNγ and IL-2 secretion was measured by ELISPOT assay. We detected IFNγ and IL-2 secreting cells against eight (Figure 1A) HIVBr18- encoded peptides; all peptides that induced IFNγ secretion were also capable to elicit IL-2 secretion. The recognized peptides p17 (73–89), p6 (32–46), pol (785–799), gp160 (188–201), rev (11027), vpr (58–65), vif (144–158) and nef (180–194) consistently presented positive responses over multiple independent immunization experiments. In contrast, T cells from pVAX1 immunized mice presented negligible numbers of IFNγ and IL-2 secreting cells after incubation with HIVBr18 peptides, in all performed experiments.

**Figure 2:**
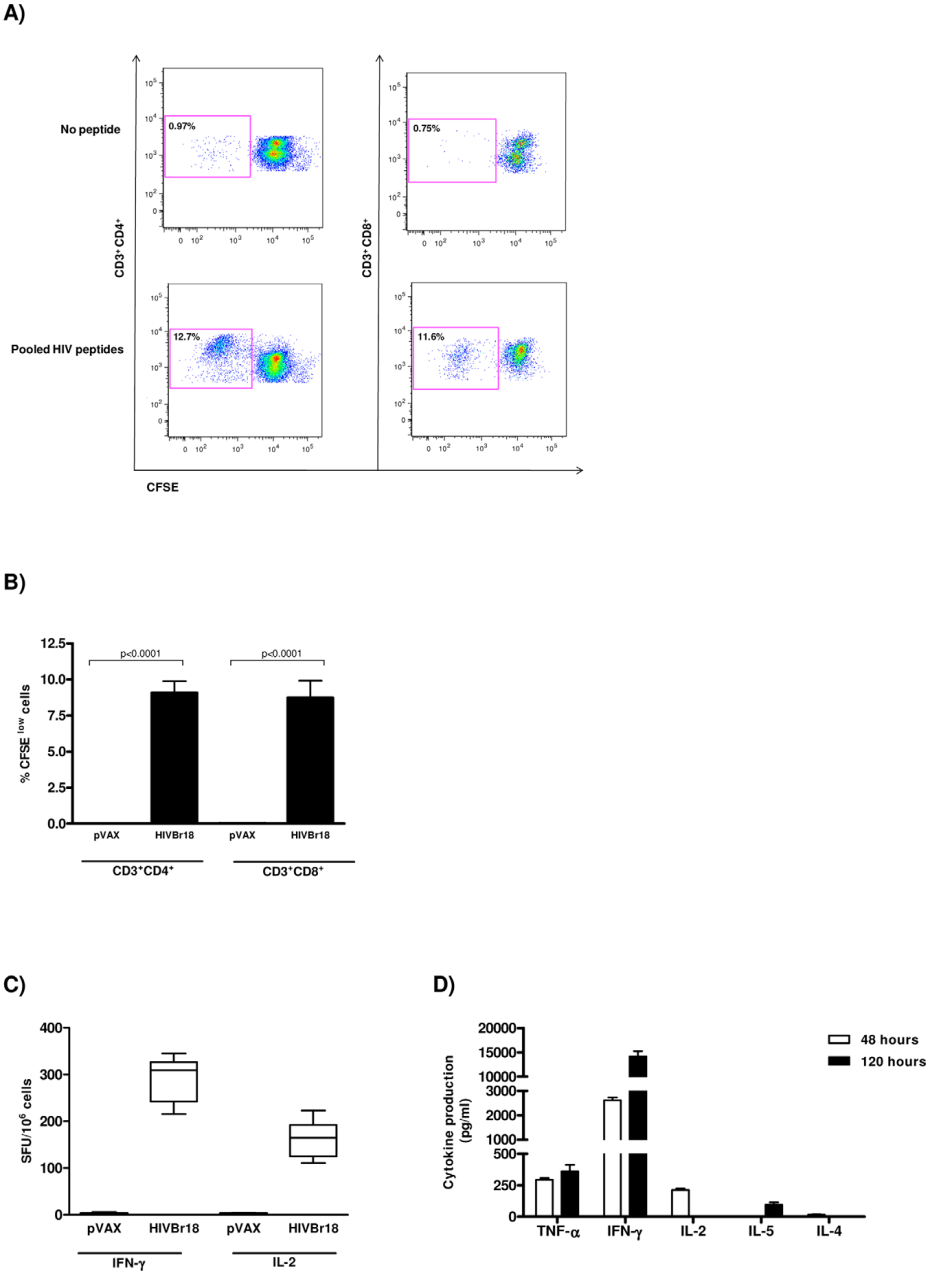
Immunization with HIVBr18 induces CD4^+^ and CD8^+^ T cell proliferation and type 1 cytokine production in response to pooled HIV-1 peptides. Two weeks after the last immunization with HIVBr18 or the empty pVAX1 vector, pooled spleen cells from 6 BALB/c mice were cultured in the presence of 5 µM of pooled HIV-1 peptides or medium only. (A and B) Splenocytes were labeled with CFSE (1.25 µM) and cultured for 5 days. After staining with fluorochrome-labeled anti-CD3, -CD4 and -CD8 monoclonal antibodies, cells were analyzed by flow cytometry and CFSE dilution on gated CD3^+^CD4^+^ or CD3^+^CD8^+^ cells was used as a readout for antigen-specific proliferation. (A) Representative dot plots of CD4^+^ (left) and CD8^+^ (right) T cell proliferation (% CFSE^low^ cells) of splenocytes stimulated with medium only or pooled peptides from HIVBr18 immunized mice. (B) Quantitative analysis of proliferating CD4^+^ and CD8^+^ CFSE^ low^ T cells (values in boxes represent % CFSE^low^ cells). The percentage of proliferating T cells was calculated subtracting the values of peptide-stimulated from non-stimulated cultures; (C) Frequencies of IFNγ and IL-2 secreting cells measured by ELISPOT; (D) Splenocytes from immunized BALB/c mice were cultured in the presence of pooled HIV-1 peptides. After 48 and 120 hours, levels of IFNγ, TNFα, IL-2, IL-4 and IL-5 in culture supernatants were measured using the mouse Th1/Th2 cytokine cytometric bead array (CBA) by flow cytometry and analyzed using CBA software. Values of cytokine production by peptide-stimulated splenocytes from pVAX1 immunized group were always below the detection limit. Bars represent the mean + 3 SD from nine independent immunization experiments.

To identify CD4^+^ and CD8^+^ T cell responses, we performed a CFSE-based proliferation assay. Taking into account multiple independent experiments, we detected consistent CD4^+^ T cell proliferative responses (Figure 2B, upper panels) against five peptides (p6 (32–46), pol (785–799), gp160 (188–201), vif (144–158) and nef (180–194)) and CD8^+^ T cell proliferative responses (Figure 2B, lower panels) against two peptides (gp160 (188–201) and nef (180–194)). Interestingly, all peptides that elicited proliferative CD4^+^ and/or CD8^+^ T cell responses were also able to elicit cytokine (IFNγ and IL-2) secretion. Thus, vaccination of BALB/c mice with HIVBr18 was able to induce broad specific immune responses against eight epitopes encoded by the vaccine.

### Magnitude of T cell responses

To assess the magnitude of vaccine-induced T cell responses, we evaluated the cellular immune responses of splenocytes from HIVBr18 or pVAX1 immunized BALB/c mice against pooled HIV-1 peptides. We observed that over 10% of both CD3^+^CD4^+^ and CD3^+^CD8^+^ splenic T cells from HIVBr18 immunized mice displayed specific proliferation against the HIV-1 peptides encoded by HIVBr18 (Figure 2A and B). In addition, BALB/c mice immunized with HIVBr18 displayed a significant number of peptide-specific IFNγ and IL-2 secreting cells (Figure 2C). In contrast, splenocytes from pVAX1 immunized mice presented negligible levels of proliferation and cytokine secreting cells to the same pooled HIV-1 peptides (Figures S1, 2B and C).

The results showed above indicate that immunization with HIVBr18 is able to induce Th1 cytokines. In order to analyze the vaccine induced cytokine secretion profile of BALB/c splenocytes incubated with the pooled HIV-1 peptides, we used the cytometric bead array (CBA) for assessment of Th1 and Th2 cytokine secretion. After 48 hours of culture, we found that splenocytes from HIVBr18 immunized mice produced higher levels of type I cytokines like IFNγ, IL-2 and TNFα and negligible levels of IL-5 and IL-4. After 120 hours of culture, the levels of IFNγ and TNFα increased substantially (Figure 2D and S2). Of note, IL-10 production was undetectable (data not shown). Splenocytes from pVAX1 immunized mice failed to secrete cytokines above the detection limit. Taken together, these results indicated that the HIVBr18 DNA vaccine induced potent, specific type 1 cytokine T cell responses.

### Functional profile of cellular immune response after HIVBr18 immunization

**Figure 3:**
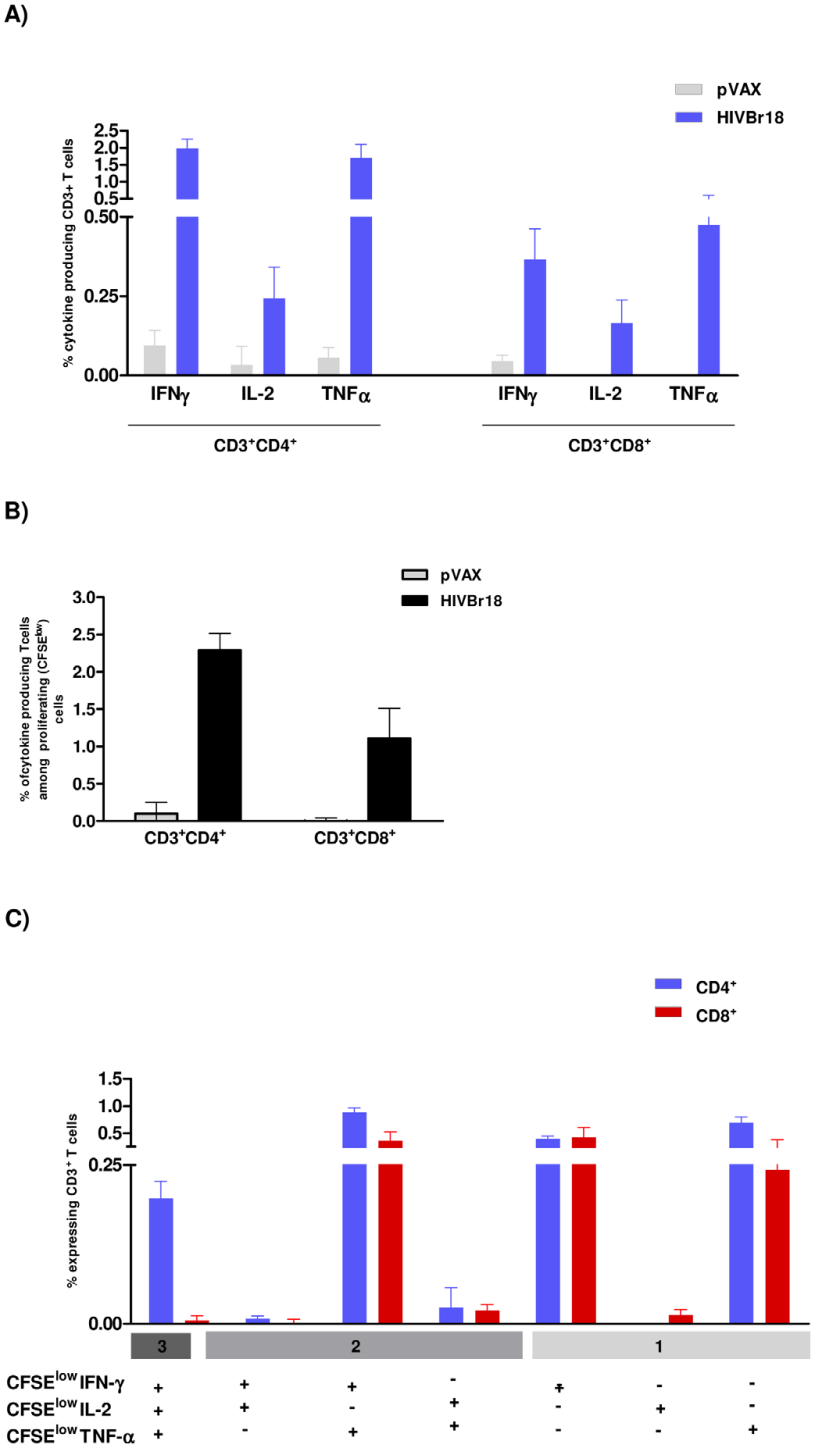
Immunization with HIVBr18 induces proliferating T cells with a polyfunctional type 1 cytokine profile. Two weeks after the last immunization with HIVBr18 or the empty vector pVAX1, spleen cells from 6 BALB/c mice were collected, labeled with CFSE (1.25 µM) and cultured for 4 days in the presence of pooled HIV-1 peptides or medium only. On day 4, cells were pulsed for 12 hours with pooled peptides in the presence of costimulatory antibody and Brefeldin A. Cells were then surface stained with antibodies to CD4 and CD8, permeabilized and stained for intracellular cytokines (IFNγ, TNFα and IL-2) and CD3. (A) Multiparameter flow cytometry was used to determine the frequency of IFNγ, IL-2 or TNFα CD4^+^ and CD8^+^ T cells. (B) Total frequencies of proliferating (CFSE^low^) and cytokine-producing CD4^+^ and CD8^+^ T cells; (C) After gating on proliferating (CFSE^low^) and cytokine-producing cells, Boolean combinations were then created using FlowJo software to determine the frequency of each response based on all possible combinations of cytokine expression. Background responses detected in negative control tubes were subtracted from those detected in stimulated samples for every specific functional combination. Negative control tubes include cells stimulated with medium and cells from pVAX1 immunized mice stimulated with pooled peptides. For each sample 500,000 events were collected in the live lymphocyte gate. Results are representative of two to three independent experiments.

Since the quality of the immune responses has been associated with vaccine-mediated protection against certain pathogens, we subsequently characterized the phenotype and functional profile of the induced T cells. Using multiparameter flow cytometry, we sought to characterize antigen-specific T cells (CD4^+^ and CD8^+^) based on their ability to proliferate (CFSE dilution assay) and produce the effector cytokines IFNγ, TNFα and IL-2 at a single cell level. As shown in Figure 3A, immunization with HIVBr18 induced HIV-1 peptide-specific production of IFNγ, IL-2 and TNFα by CD4^+^ and to a lesser extent by CD8^+^ T cells. We also observed that both T cell subsets showed a higher proportion of of IFNγ^+^ and TNFα^+^ cells than IL-2^+^ cells. A simultaneous analysis of proliferation and intracellular cytokine production demonstrated that 2.3% of CD4^+^ and 1.0% of CD8^+^ T cells proliferated (CFSE^low^) and produced any cytokine tested in response to pooled HIV-1 peptides (Figure 3B). Boolean combinations of proliferating (CFSE^low^) and cytokine-positive populations indicated that HIVBr18 immunization induced polyfunctional CD4^+^ and CD8^+^ T cells, i.e., that proliferate (CFSE^low^) and produce IFNγ/TNFα simultaneously (Figure 3C). We also observed high proportions of CFSE^low^/IFNγ or CFSE^low^/TNFα in CD4^+^ and CD8^+^ T cells from mice that received HIVBr18. For CD4^+^ T cells exclusively, we also detected CFSE^low^ cells that simultaneously produced all three tested cytokines (IFNγ/TNFα/IL-2). Splenocytes from the pVAX1 immunized group produced negligible levels of cytokines. Furthermore, triple-cytokine producing cells produced more IFNγ and TNFα than single-cytokine producing cells, as determined by median fluorescent intensity (MFI) (Figure S3). In contrast, there was no difference in the MFI for IL-2 in the triple-cytokine producing cells when compared to cells producing IL-2 alone.

**Figure 4:**
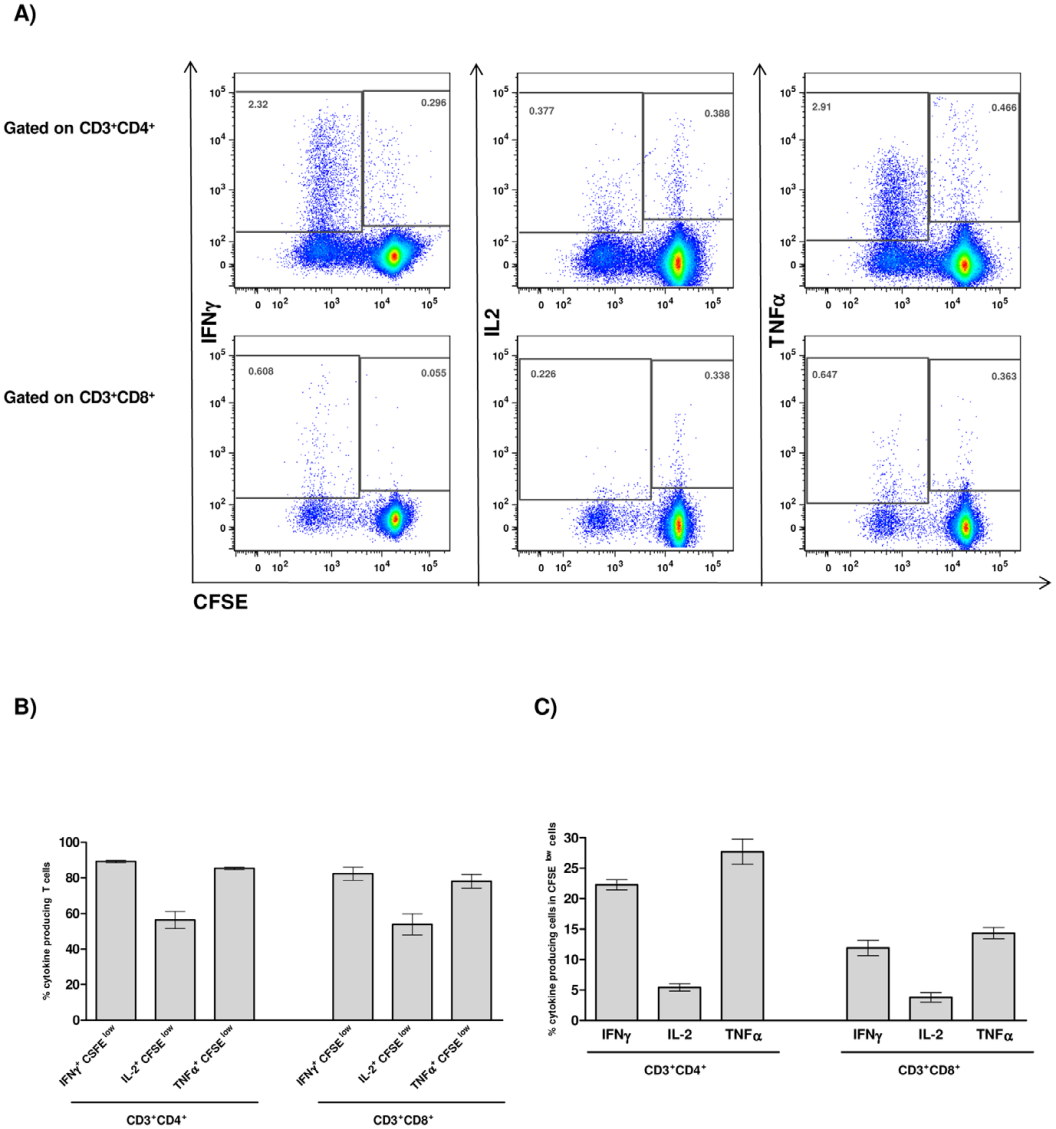
Immunization with HIVBr18 induces specific CD4^+^ and CD8^+^ T cells that proliferate and produce type 1 cytokines simultaneously. Two weeks after the last immunization with HIVBr18 or the empty vector pVAX1, spleen cells from 6 BALB/c mice were labeled with CFSE (1.25 µM) and cultured for 4 days in the presence of pooled HIV-1 peptides or medium only. On day 4, cells were pulsed for 12 hours with pooled peptides or medium, in the presence of costimulatory antibody and Brefeldin A. (A) CFSE and intracellular cytokine staining were used to simultaneously assess proliferation and IFNγ, TNFα or IL2 production. Frequencies of antigen-specific cytokine-producing T cells in proliferating (CFSE^low^) and non-proliferating (CFSE^hi^) gates are displayed; (B) Proportion of proliferating cells (CFSE^low^) in the total cytokine gate (sum of % of cytokine producing cells in gated CFSE^hi^ and CFSE^low^ cells); (C) Percentage of cytokine producing CD4^+^ and CD8^+^ T cells in proliferating cells (CFSE^low^) (values of cytokine producing cells in CFSE^low^ population ×100 divided by total CFSE^low^ population).

We next examined whether antigen-specific proliferating T cells were the major cytokine producers. As shown in Figures 4A and B, the vast majority (ca. 80%) of CD4^+^ and CD8^+^ T cells that produced the effector cytokines IFNγ and TNFα are within the proliferating (CFSE^low^) population. In contrast, 50% of IL-2 producing T cells also proliferated (Figure 4B). These experiments also showed that mice immunized with HIVBr18 displayed 2.31, 0.37 and 2.80% of HIV-specific CD4^+^ T cells that proliferated (CFSE^low^) and produced either IFNγ, IL-2 or TNFα, respectively. A similar response was observed in CD8^+^ T cells, showing that 0.60, 0.22 and 0.64% of CD8^+^ T cells proliferated and produced either IFNγ, IL-2 or TNFα, respectively. In contrast, splenocytes from mice immunized with the control pVAX1 displayed a negligible percentage of specific proliferating/cytokine producing T cells (Figure S4).

Proliferating (CFSE^low^) and non-proliferating (CFSE^hi^) CD4^+^ and CD8^+^ T cells were also evaluated by their ability to produce cytokines. Figure 4C summarizes the percentage of proliferating T cells that produced each of the tested cytokines. Significantly, 20% of the specific proliferating CD4^+^ T cells produced IFNγ, 5% produced IL-2 and 30% produced TNFα. A similar profile, albeit with lower values, was observed for proliferating CD8^+^ T cells. In contrast, less than 0.5% of non-proliferating (CFSE^hi^) T cells showed cytokine production, either on CD4^+^ or CD8^+^ compartment. Overall, these data showed that immunization with the DNA vaccine, HIVBr18, successfully induced polyfunctional CD4^+^ and CD8^+^ T cells that proliferated and produced effector cytokines to epitopes encoded by the vaccine.

### Induction of long-lasting HIV-1-specific T cells after vaccination with HIVBr18

**Figure 5:**
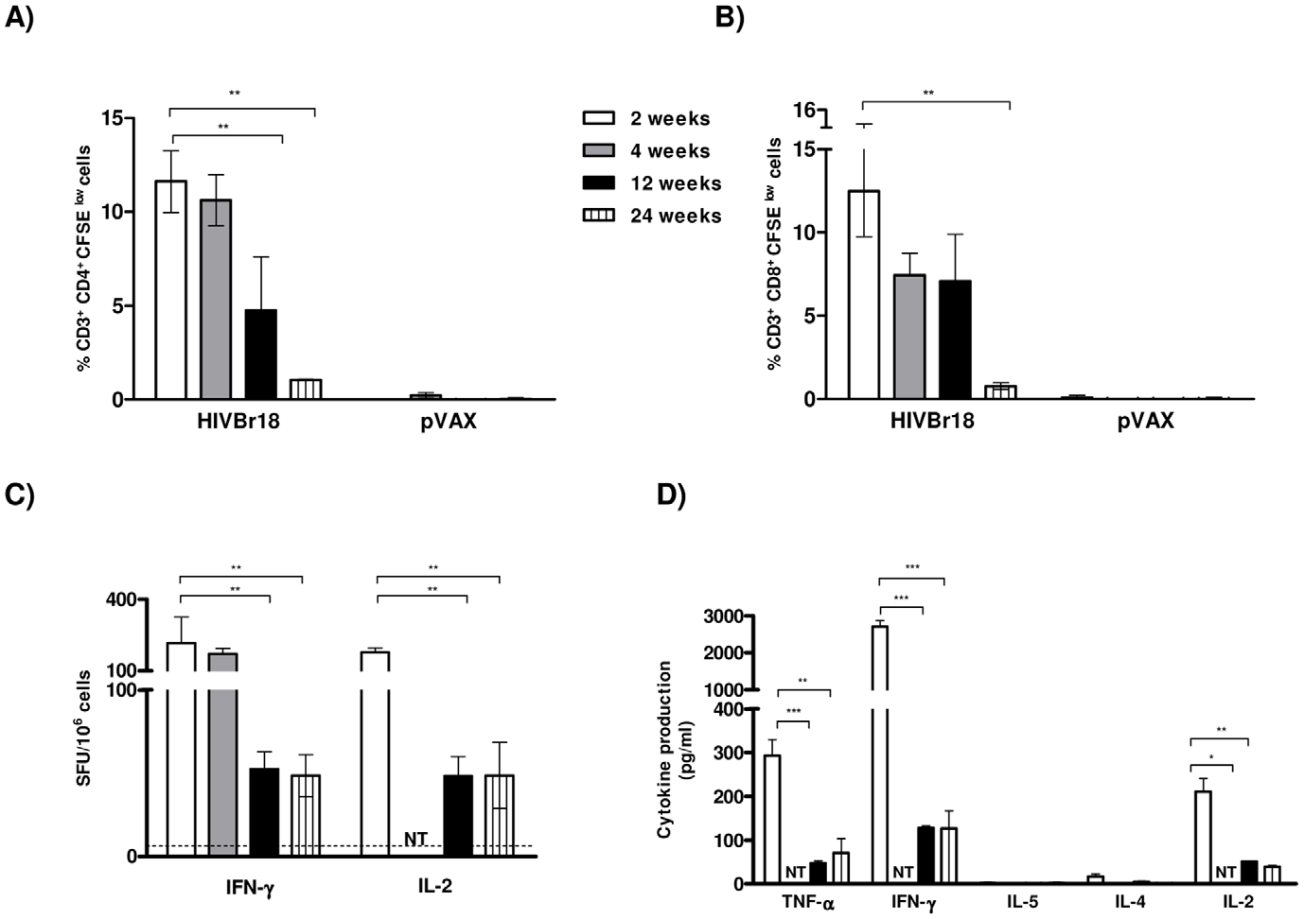
Longevity of the antigen specific cellular immune response induced by HIVBr18. BALB/c mice immunized with HIVBr18 were euthanized at the indicated time points after the last immunization. Splenocytes were cultured in the presence of pooled HIV-1 peptides. Percentage of proliferating CD3^+^CD4^+^ (A) and CD3^+^CD8^+^ (B) T cells, 2 (white bar), four (dark gray bar), twelve (black bar) and 24 weeks (striped bar) after last injection. Values of proliferating (CFSE^low^) CD3^+^CD4^+^ T cells from the pVAX1 group after background subtraction were 0.11%; 0.24%; 0% and 0.03% at 2, 4, 12 and 24 weeks respectively. Values of CD3^+^CD8^+^ CFSE^low^ cells were always below 0.1%; (C) Frequencies of IFNγ and IL-2- secreting cells as measured by ELISPOT assay. Values from the pVAX1 immunized group were always below 5 SFU/10^6^ cells; (D) After 48 hours of incubation with pooled HIV-1 peptides, culture supernatants were analyzed for the presence of IFNγ, TNFα, IL-2, IL-4 and IL-5 using the mouse Th1/Th2 cytokine cytometric bead array (CBA). NT = not tested; *p<0.05, **p<0.01, ***p<0.001.

To assess whether immunization with HIVBr18 induced long-lasting T cells in BALB/c mice, we measured vaccine-induced CD4^+^ and CD8^+^ T cell proliferation and cytokine secretion 2, 4, 12 and 24 weeks after the last DNA immunization. A measurable response was observed at all time points. At 2 and 4 weeks post-immunization, a similar proportion of CD4^+^ T cells (11–12%) proliferated against the pooled HIV-1 peptides (Figure 5A). Twelve weeks after the last dose, a statistically significant decrease in the magnitude of the CD4^+^ T cell proliferative response was observed (4.76% CFSE^ low^ cells, versus 11.62% in early time points, p<0.01). This response continued to decline down to 1% of specific proliferating CD4^+^ T cells, 24 weeks after the last dose. Of note, this response was several-fold higher than the values measured in the pVAX1 immunized group. For CD8^+^ T cells (Figure 5B), only the 24 week time point showed a statistically significant decrease in proliferation, as compared to the 2 week time point. Splenic T cells from mice immunized with pVAX1 showed negligible levels of proliferation at all time points for both CD4^+^ and CD8^+^ populations (values in the legend of Figure 5A and B). Of note, 24 weeks after the last immunization, total frequencies of proliferating CD4^+^ and CD8^+^ T cells were ca. 1% above baseline, as well as above the values from the pVAX1 immunized group. The ability of T cells from HIVBr18 immunized mice to secrete cytokines was also evaluated by ELISPOT (Figure 5C) and CBA (Figure 5D) assays at different time points. As observed for proliferative responses, there was no significant difference in IFNγ secretion to pooled HIV-1 peptides at 2 or 4 weeks after the last HIVBr18 immunization. IFNγ secretion decreased significantly (p<0.01) 12 weeks after the last dose and was sustained until the 24 week time point (Figure 5C). Similar results were observed when we measured IL-2 secretion (Figure 5C). Splenocytes from HIVBr18 immunized mice were able to secrete type I cytokines like IFNγ, IL-2 and TNFα, which declined over time, as evaluated by the CBA assay, but were still detectable and above pVAX1 immunized group, even 24 weeks after the last dose (Figure 5D). Therefore, long-term proliferative CD4^+^ and CD8^+^ T cell responses as well as cytokine-secreting responses were detectable up to 24 weeks after the last immunization with HIVBr18.

### HIVBr18 immunization induces long-term memory T cells

In order to characterize memory T cell responses induced by immunization, we assessed the expression of memory markers (CD44 and CD62L) among proliferating (CFSE^low^) CD4^+^ and CD8^+^ T cell populations. We evaluated responses against pooled HIV-1 peptides, 2 weeks after the last immunization with HIVBr18. The candidate vaccine HIVBr18 elicited higher levels of specific effector (T_EM_) than central (T_CM_) memory T cells. Among proliferating CFSE^low^ CD4^+^ T cells (8% of total CD4^+^ T cells), ca. 70% and 24% had effector and central memory phenotypes, respectively (data not shown). Similarly, 57% and 39% of proliferating CD8^+^ T cells (7% of total CD8^+^ T cells) had effector and central memory phenotypes, respectively (data not shown).

**Figure 6:**
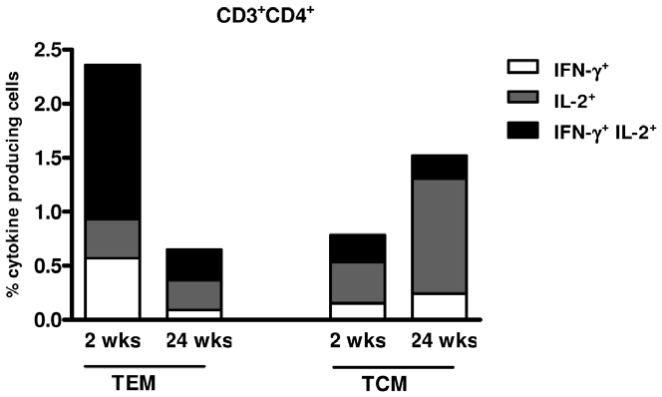
Memory T cell kinetics after immunization with HIVBr18. Two and 24 weeks after the last immunization with HIVBr18 or the empty vector pVAX1, spleen cells from 6 BALB/c mice were cultured in the presence of pooled HIV-1 peptides. Simultaneous assessment of antigen-specific cytokine production and T cell memory phenotype was performed in gated CD3^+^CD4^+^ cells. Analysis of CD44 and CD62L expression was determined on gated CD3^+^CD4^+^ cells. IFNγ and/or IL-2 producing effector (CD44^hi^ CD62L^low^) and central memory (CD44^hi^ CD62L^hi^) CD4^+^ T cells are depicted.

We also investigated the phenotype of specific cytokine-producing CD4^+^ T cells at two time points. Two weeks after the last immunization, the most abundant population was T_EM_ cells in which more than half produced IFNγ/IL-2 simultaneously (Figure 6A). CD4^+^ T_CM_ predominantly produced IL-2 alone. The kinetic of CD4^+^Tcells demonstrated that the frequency of cytokine-producing CD4^+^ T_EM_ declined over time (2.5% at 2 weeks versus 0.65% at 24 weeks, IFNγ and/or IL-2 cells) (Figure 6A). On the other hand, cytokine-producing CD4^+^ T_CM_, especially the IL-2 component, showed an increase over time (0.8% at 2 weeks versus 1.52% at 24 weeks, IFNγ and/or IL-2 cells). Collectively, these data indicated that the DNA vaccine HIVBr18 was able to induce specific long-lasting effector and central memory, CD4^+^ and CD8^+^ T cells that produced type 1 cytokines.

## Discussion

In this paper, we showed that immunization with HIVBr18 induced strong, broad, polyfunctional and long-lasting HIV-specific CD4^+^ and CD8^+^ T cell responses in BALB/c mice. Moreover, the vaccine induced central and effector memory CD4^+^ T cells.

In our study, all the 18 TEPITOPE-selected promiscuous epitopes were also predicted by the algorithm PRED^BALB/c^ [36] to bind to at least one BALB/c (H-2^d^) mouse MHC class II molecule, and 17 were predicted to bind to H-2^d^ MHC class I molecules. The cross-species recognition of epitopes selected to bind to human MHC molecules is actually expected. The TEPITOPE algorithm has previously identified peptides that can be recognized across species barriers, including H-2^d^ mice [37]. This may be due to the fact that the identification of multiple HLA-DR binding peptides by TEPITOPE may select for promiscuous peptides that share MHC class II binding motifs similar to many other human and non-human MHC class II molecules [38]–[40]. Indeed, we also tested this concept in non-human primates. TEPITOPE selection for promiscuous peptides allowed the identification of a similar set of conserved SIV peptides that were frequently recognized by PBMC from SIV-infected elite controller rhesus macaques (unpublished data). Here, we have shown that BALB/c mice immunized with HIVBr18 presented a broad T cell response, directed to 8 out of the 18 epitopes encoded by the vaccine. The induction of broad T cell responses towards conserved epitopes appears to be an essential pre-requisite for protection, in light of recent efficacy trials of T cell-based HIV vaccines [1], [4], [5]. Indeed, an Adenovirus 5-based SIV vaccine encoding 8 SIV proteins was able to elicit broad CD4^+^ and CD8^+^ T cell responses and reduced viral load after heterologous challenge [6]. In addition, broad vaccine-induced pre-challenge T cell responses were correlated with lower viral loads and higher CD4^+^ T lymphocyte counts [41]. The breadth of HIV-specific responses in infected individuals has also been associated with viral control [42].

We also evaluated the functional profile of the vaccine-induced specific CD4^+^ and CD8^+^ T cells. The ability of the HIVBr18 vaccine to induce IFNγ, TNFα and IL-2, with little or no IL-4, IL-5 or IL-10, indicated a type 1 cytokine response. We extended the functional analysis of splenocytes from HIVBr18 immunized mice and observed that immunization induced CD4^+^ and CD8^+^ T cells with a polyfunctional repertoire. It has become increasingly evident that rather than the magnitude, the quality of the immune response is a crucial factor in defining a protective response [28]. Recent data from immunization studies using the efficacious smallpox and yellow fever vaccines have shown that induction of specific polyfunctional CD4^+^ or CD8^+^ T cells may play a role in protective immunity [43]–[45]. Furthermore, vaccine-induced polyfunctional (IFNγ^+^IL-2^+^TNFα^+^) CD4^+^ T cell populations were shown to provide protection against *Leishmania major* [46] and *M. tuberculosis* [47] challenge. Polyfunctional HIV-1-specific CD4^+^ T cells were present among HIV-1 infected individuals with non-progressive disease [24], [25]. Long-term nonprogressor (LTNP) HIV-1 infected patients displayed higher number of polyfunctional CD8^+^ T cells when compared to progressor patients [48]. Moreover, HIV elite controllers presented strong polyfunctional CD4^+^ and CD8^+^ T cell responses in blood and rectal mucosa [14], [32]. CD4^+^ T cells from HIV-2 infected subjects, who display a longer time of progression to AIDS than HIV-1 infected patients, were more polyfunctional than those found in viral load and CD4^+^ T cell-matched HIV-1 infected subjects [49]. Our data thus suggest that HIVBr18, like some validated efficacious vaccines, can elicit polyfunctional T cells, a putative correlate of protection.

Memory T cells may be critical to generate long-term immunity and to effect vaccine-induced viral control. We found that after HIVBr18 immunization, HIV-1-specific proliferating CD4^+^ T cells exhibited mainly a T_EM_ phenotype, with simultaneous IFNγ and IL-2 production, while T_CM_ cells produced mainly IL-2 alone. Specific proliferative, intracellular cytokine, and ELISPOT responses peaked 2–4 weeks after the last dose and then contracted, establishing a long-lived memory cell pool detectable until 24 weeks after the last immunization, a necessary step for protective immunity [50]. We observed that cytokine-producing HIV-1-specific CD4^+^ T_EM_ were abundant at 2 weeks but declined over time, while CD4^+^ T_CM_ increased, indicating that HIV-1-specific CD4^+^ T_CM_ induced by HIVBr18 are especially long-lived and may be able to provide sustained help to CD8^+^ T cells. Taken together, our experiments demonstrated that immunization with HIVBr18 induced long-lived CD4^+^ T cell responses with a significant T_CM_ component. Central memory T cells are thought to ensure the long-term maintenance of antiviral responses due to their long half-life and self-renewal capacity [33]. Immunization with the effective vaccinia virus is able to generate specific long-lived memory CD4^+^ T cells for more than 30 years [35]. Among HIV-1 infected LTNP individuals, there is a preserved CD4^+^ T_CM_ compartment and signs of potent functional activation in the T_EM_ CD4^+^ T cell compartment [34]. Vaccination that preserved memory CD4^+^ T cells in primates challenged with SIV [51] led to an increased survival [52], [53]. This reinforced the importance of memory CD4^+^ T cells in protection against AIDS progression. Further in support of this concept, vaccine- induced SIV-specific CD4^+^ and CD8^+^ T_EM_ cell responses, in the absence of neutralizing antibodies, was able to prevent the establishment of progressive systemic infection after mucosal challenge with a highly pathogenic SIV [21].

We hereby demonstrated that immunization with HIVBr18, a DNA plasmid encoding a string of conserved multiple HLA class II-binding HIV-1 CD4^+^ T cell epitopes, can induce broad, polyfunctional, long-lived CD4^+^ and CD8^+^ T cell responses. Moreover, these CD4^+^ T cell responses had significant central and effector memory components. We believe the combined administration of this vaccine concept may provide sustained help for CD8^+^ T cell –as well as antibody responses- elicited by other AIDS vaccines.

## Methods

### Construction of DNA plasmid encoding multiple HIV-1 epitopes

We designed a multiepitope construct containing the mammalian codon optimized nucleotide sequences of the 18 HIV-1 CD4 epitopes described previously by Fonseca *et al*. [26]: p17(73–89), p24 (33–45), p24 (131–150), p6 (32–46), pol (63–77), pol (136–150), pol (785–799), gp41(261–276), gp160 (19–31), gp160 (174–185), gp160 (188–201), gp160 (481–498), rev (11–27), vpr (58–72), vpr (65–82), vif (144–158), vpu (6–20) and nef (180–194). Epitope sequences, assembled *in tandem* in the above mentioned order, had GPGPG spacers at C and N termini, to avoid the creation of junctional epitopes and interference with processing and presentation [54]. The artificial gene (EZBiolab) was cloned into the HindIII/XhoI restriction site of the pVAX1 vector (Invitrogen) to generate the HIVBr18 plasmid, as previously described [27]. DNA vaccine was purified using the Endofree Plasmid Giga Kit from Qiagen according to manufacturer's instructions.

### Ethics Statement

Mice were housed and manipulated under SPF conditions in the animal care facilities of the Institute of Tropical Medicine, University of São Paulo (IMT/FMUSP). Experiments were performed in accordance to the guidelines of the Ethics committee of University of São Paulo (CAPPesq- HCFMUSP) and approved under protocol number 775-06.

### Mice and Immunizations

Six to eight week-old female BALB/c mice were used in this study. Mice were housed and manipulated under SPF conditions in the animal care facilities of the Institute of Tropical Medicine, University of São Paulo (IMT/FMUSP). Six mice per group were injected with 10 mM cardiotoxin (Sigma) five days before immunization. At weeks 0, 2 and 4, plasmid DNA HIVBr18 or empty vector pVAX1 was administered intramuscularly. Each quadriceps was injected with 50 µL of DNA at a concentration of 1 µg/µL in saline such that each animal received a total of 100 µg of plasmid DNA. Two weeks after the last DNA injection, mice were euthanized with CO_2_.

### Peptides

The eighteen multiple HLA-DR binding, frequently recognized peptides, derived from the conserved regions of HIV-1 B subtype consensus and selected from the whole proteome [26] were synthesized in house by solid phase technology using the 9-fluorenylmethoxycarbonyl (Fmoc) strategy, with the C- terminal carboxyl group in amide form [55]. Peptide purity and quality were assessed by reverse-phase high performance liquid chromatography and mass spectrometry and was routinely above 90%.

### Spleen cell isolation for immune assays

Two weeks after the last immunization, mice were euthanized and spleens were removed aseptically. After obtaining single cell suspensions, cells were washed in 10 mL of RPMI 1640. Cells were then resuspended in R-10 (RPMI supplemented with 10% of fetal bovine serum (GIBCO), 2 mM L-glutamine (Sigma), 10 mM Hepes (Sigma), 1 mM sodium piruvate, 1% vol/vol non-essential aminoacid solution, 40 µg/mL of Gentamicin, 20 µg/mL of Peflacin and 5×10^−5^ M 2- mercaptoetanol (SIGMA). The viability of cells was evaluated using 0.2% Trypan Blue exclusion dye to discriminate between live and dead cells. Cell concentration was estimated with the aid of a Neubauer chamber and adjusted in cell culture medium.

### Detection of IFNγ or IL-2 producing cells by ELISPOT assay

Splenocytes from HIVBr18 or pVAX1 immunized mice were assayed for their ability to secrete IFNγ or IL-2 after *in vitro* stimulation with 5 µM of individual or pooled HIV-1 peptides using an ELISPOT assay. The ELISPOT assay was performed using Becton Dickinson murine IFNγ and IL-2 ELISPOT kits according to manufacturer's instructions. Spots were counted using an automated stereomicroscope (KS ELISPOT, Zeiss, Oberkochem, Germany). The number of antigen- specific T cells, expressed as spot-forming units (SFU)/10^6^ splenocytes, was calculated after subtracting negative control values (medium only). The positivity cutoff was calculated as the mean + 3 SD of splenocytes from pVAX1 immunized mice, stimulated with all peptides. The cutoff for IFNγ and IL-2 was 15 SFU/10^6^ splenocytes.

### Cytometric Bead array

One million splenocytes were added to each well of 96 well round-bottomed plates and incubated at indicated time points in the presence 5 µM of pooled HIV-1 peptides. Supernatants harvested from cultures at the indicated times were stored at −20°C until cytokine analysis was performed. IL-2, IL-4, IL-5, TNFα and IFNγ were detected simultaneously using the mouseTh1/Th2 cytokine cytometric bead array (CBA) kit (BD PharMingen), according to the manufacturer's instructions. The range of detection was 20–5000 pg/mL for each cytokine.

### CFSE-based proliferation assay

To monitor the expansion and proliferation of HIV-specific T cells, splenocytes from HIVBr18 or pVAX1 immunized mice were labeled with carboxyfluorescein succinimidyl ester (CFSE) [56]. Briefly, freshly isolated splenocytes were resuspended (50×10^6^/mL) in PBS and labeled with 1.25 µM of CFSE (Molecular Probes) at 37°C for 10 minutes. The reaction was quenched with RPMI 1640 supplemented with 10% FBS and cells were washed before resuspending in RPMI 1640 at a density of 1.5×10^6^/mL. Cells were cultured in 96 well round-bottomed plates (3×10^5^/well in triplicate) for 5 days at 37°C and 5% CO_2_ with medium only or 5 µM of HIV peptides. Positive controls were stimulated with 2.5 µg/mL of Concanavalin A (Sigma). Cells were then harvested, washed with 100 µL of FACS buffer (PBS with 0.5% BSA and 2 mM EDTA) and stained with anti-mouse CD3 phycoerythrin (PE), anti-mouse CD4 peridinin chlorophyll protein (PerCP) and anti-mouse CD8 allophycocyanin (APC) monoclonal antibodies (BD Pharmingen, San Jose, CA) for 45 minutes at 4°C. To analyze the memory phenotype of proliferating cells we stained the cells with anti-CD3 APCCy7, anti-CD4 PerCP, anti-CD8 PECy7, anti-CD44 PE and anti-CD62L APC monoclonal antibodies (BD Pharmingen). Cells were then washed twice with FACS buffer, fixed with 4% paraformaldehyde, and resuspended in FACS buffer. Samples were acquired on a FACSCanto flow cytometer (BD Biosciences) and then analyzed using FlowJo software (version 9.0.2, Tree Star, San Carlo, CA). Fifty thousand events (proliferation evaluation) and 100,000 events (memory phenotype) were acquired in a live lymphocyte gate. The percent of proliferating CD4 ^+^ and CD8^+^ CFSE^low^ cells was determined in the CD3^+^ cell population. The criteria for scoring as positive the proliferating cell cultures included CFSE^low^ cells > cutoff. The cutoff of unspecific proliferative response was determined based on the median percentage of proliferating cells (% of CD3^+^CD4^+^ or CD3^+^CD8^+^ CFSE^low^ cells) on splenocytes from pVAX1 immunized groups after stimulating with individual peptides +3 standard deviation (SD).

### Analysis of polyfunctional HIV-specific T cell responses

Splenocytes from immunized mice were labeled with CFSE as described above. CFSE-labeled cells were incubated at a density of 2.5×10^6^ cells/mL and cultured in 96 well round-bottomed plates (5×10^5^/well in triplicate) for 4 days at 37°C and 5% CO_2_ with medium only or pooled HIV peptides (5 µM). After 4 days of incubation, cells were restimulated in the presence of 2 µg/mL anti-CD28 (BD Pharmingen), 5 µM of pooled HIV peptides and Brefeldin A- GolgiPlug™ (BD Pharmingen) for the last 12 hours. After the incubation period, cells were washed with FACS buffer and surface stained using monoclonal antibodies to CD8-Alexa700 and CD4-PerCP for 30 minutes at 4°C. Cells were fixed and permeabilized using the Cytofix/Cytoperm™ kit (BD Pharmingen). Permeabilized cells were washed with Perm/Wash buffer (BD Biosciences) and stained with monoclonal antibodies to CD3-APCCy7, IL2-PE, TNFα-PECY7 and IFNγ-APC for 30 minutes at 4°C. Following staining, cells were washed twice and resuspended in FACS buffer. All antibodies were from BD Pharmingen. Samples were acquired on a FACSCanto flow cytometer (BD Biosciences) and then analyzed using FlowJo software (version 9.0.2, Tree Star, San Carlo, CA). Each analysis was gated on forward (FSC)/side scatter (SSC) lymphocytes (500,000 events) and CD3^+^ T cells followed by a subsequent gate on CD4^+^ or CD8^+^ populations. After identification of CD4^+^ and CD8^+^ populations, a gate was done in each positive population for IFNγ, TNFα and IL-2 expression. In addition, we used the Boolean gate (FlowJo software (version 9.0.2, Tree Star, San Carlo, CA)) platform to create several combinations of the three cytokine (IL-2, TNFα and IFNγ) within CFSE^low^ population resulting in seven distinct patterns. The percentages of cytokine-producing cells were calculated by subtracting background values. For each flow cytometry experiment performed in this paper, unstained and all single-color controls were processed to allow proper compensation.

### Data Analysis

Statistical significance (p-values) was calculated by using One-way ANOVA and Tukey's honestly significantly different (HSD). Statistical analysis and graphical representation of data was performed using GraphPad Prism version 5.0 software.

## Supporting Information

Figure S1 **Proliferative responses of CD4^+^ and CD8^+^ T cells from pVAX1 immunized mice against pooled HIV-1 peptides.** BALB/c mice were immunized with the empty vector pVAX1. Two weeks after the last dose, pooled spleen cells from 6 mice were labeled with CFSE (1.25 µM) and cultured for 5 days in the presence of 5 µM of pooled HIV-1 peptides. Cells were analyzed by flow cytometry and CFSE dilution on gated CD3^+^CD4^+^ or CD3^+^CD8^+^cells was used as a readout for antigen-specific proliferation. Representative dot plots of CD4^+^ (left) and CD8^+^ (right) T cell proliferation (% CFSE^low^ cells) from splenocytes stimulated with medium or pooled HIV-1peptides. Data are representative of nine independent immunization experiments.
(TIF)

Figure S2 **Immunization with HIVBr18 induces a HIV-1 peptide-specific type 1 cytokine response.** Splenocytes from immunized BALB/c mice were cultured in the presence of pooled HIV-1 peptides. After 48 hours, levels of IFNγ, TNFα, IL-2, IL-4 and IL-5 in culture supernatants were measured using the mouse Th1/Th2 cytokine cytometric bead array (CBA) by flow cytometry. Representative dot plot profiles of the 6-plex Th1/Th2 cytokine CBA assay for culture supernatants from pVAX1 (left) and HIVBr18 (right) immunized mice after stimulation with pooled HIV-1 peptides.
(TIF)

Figure S3 **Polyfunctional CD4^+^ T cells produce higher cytokine levels on a per cell basis than single cytokine-producing CD4^+^ T cells.** Two weeks after the last immunization with HIVBr18, spleen cells from 6 BALB/c mice were labeled with CFSE and cultured in the presence of pooled HIV-1 peptides or medium only for 4 days. On day 4, cells were pulsed for 12 hours with pooled HIV-1 peptides or medium in the presence of costimulatory antibody and Brefeldin. Multiparameter flow cytometry was used to identify polyfunctional and single cytokine-producing CD3^+^CD4^+^ T cells. Intracellular cytokine levels expressed as MFI values are compared for CFSE^low^ cells producing all 3 tested cytokines (polyfunctional cells) and CFSE^low^ cells producing a single cytokine. MFI values for IFNγ (*left*),TNFα (*middle*) and IL-2 (*right*).
(TIF)

Figure S4 **Proliferative responses and cytokine production in splenocytes from pVAX1 immunized mice.** Two weeks after the last immunization with the empty vector pVAX1, spleen cells from 6 BALB/c mice were labeled with CFSE and cultured in the presence of pooled HIV-1 peptides or medium only for 4 days. On day 4, cells were pulsed for 12 hours with pooled HIV-1 peptides or medium in the presence of costimulatory antibody and Brefeldin A. CFSE and intracellular cytokine staining were used to simultaneously assess proliferation and IFNγ, TNFα or IL-2 production. Frequencies of antigen-specific cytokine-producing T cells in proliferating (CFSE^low^) and non proliferating (CFSE^hi^) gates are displayed.
(TIF)

Table S1 **Peptide binding predictions for H-2d MHC class I and class II.**
(PDF)
