# Supplementary figures and images for: A DNA Vaccine Encoding Multiple HIV CD4 Epitopes Elicits Vigorous Polyfunctional, Long-Lived CD4+ and CD8+ T Cell Responses
Source: PLoS One. 2011 Feb 11;6(2):e16921. doi: 10.1371/journal.pone.0016921 (PMC3037933; doi:10.1371/journal.pone.0016921)

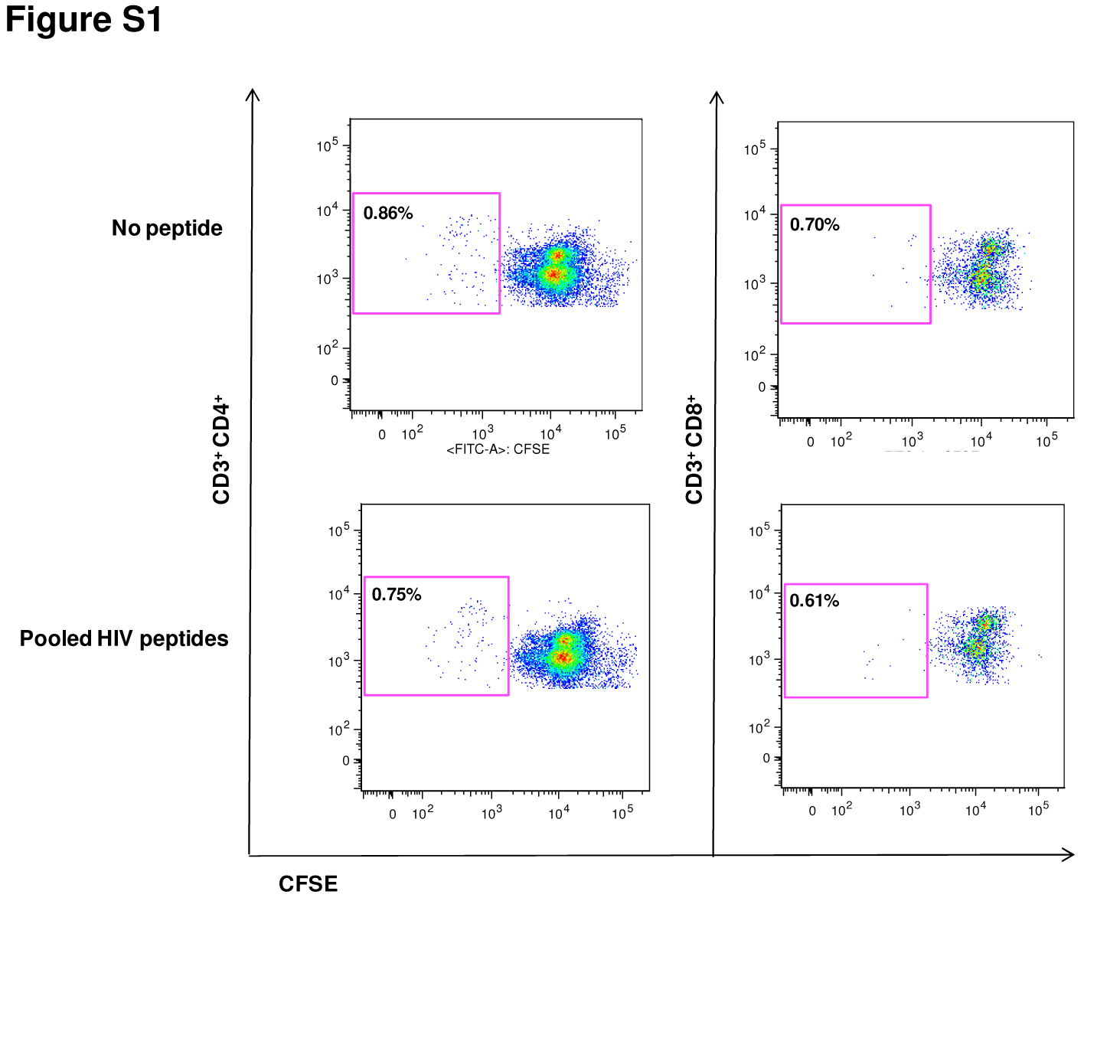

Supplement: Figure S1 — Proliferative responses of CD4+ and CD8+ T cells from pVAX1 immunized mice against pooled HIV-1 peptides. BALB/c mice were immunized with the empty vector pVAX1. Two weeks after the last dose, pooled spleen cells from 6 mice were labeled with CFSE (1.25 µM) and cultured for 5 days in the presence of 5 µM of pooled HIV-1 peptides. Cells were analyzed by flow cytometry and CFSE dilution on gated CD3+CD4+ or CD3+CD8+cells was used as a readout for antigen-specific proliferation. Representative dot plots of CD4+ (left) and CD8+ (right) T cell proliferation (% CFSElow cells) from splenocytes stimulated with medium or pooled HIV-1peptides. Data are representative of nine independent immunization experiments. (TIF) [file pone.0016921.s001.tif]

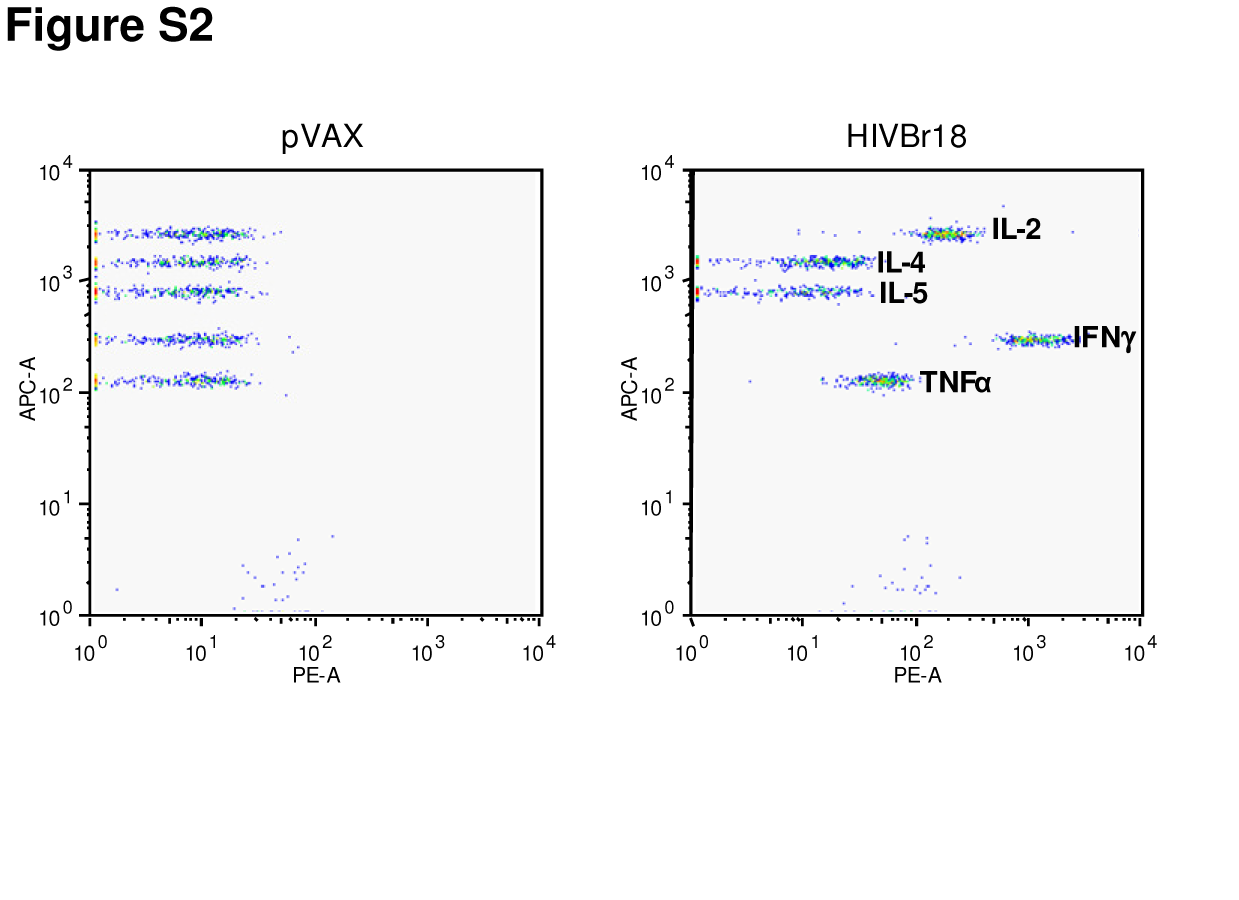

Supplement: Figure S2 — Immunization with HIVBr18 induces a HIV-1 peptide-specific type 1 cytokine response. Splenocytes from immunized BALB/c mice were cultured in the presence of pooled HIV-1 peptides. After 48 hours, levels of IFNγ, TNFα, IL-2, IL-4 and IL-5 in culture supernatants were measured using the mouse Th1/Th2 cytokine cytometric bead array (CBA) by flow cytometry. Representative dot plot profiles of the 6-plex Th1/Th2 cytokine CBA assay for culture supernatants from pVAX1 (left) and HIVBr18 (right) immunized mice after stimulation with pooled HIV-1 peptides. (TIF) [file pone.0016921.s002.tif]

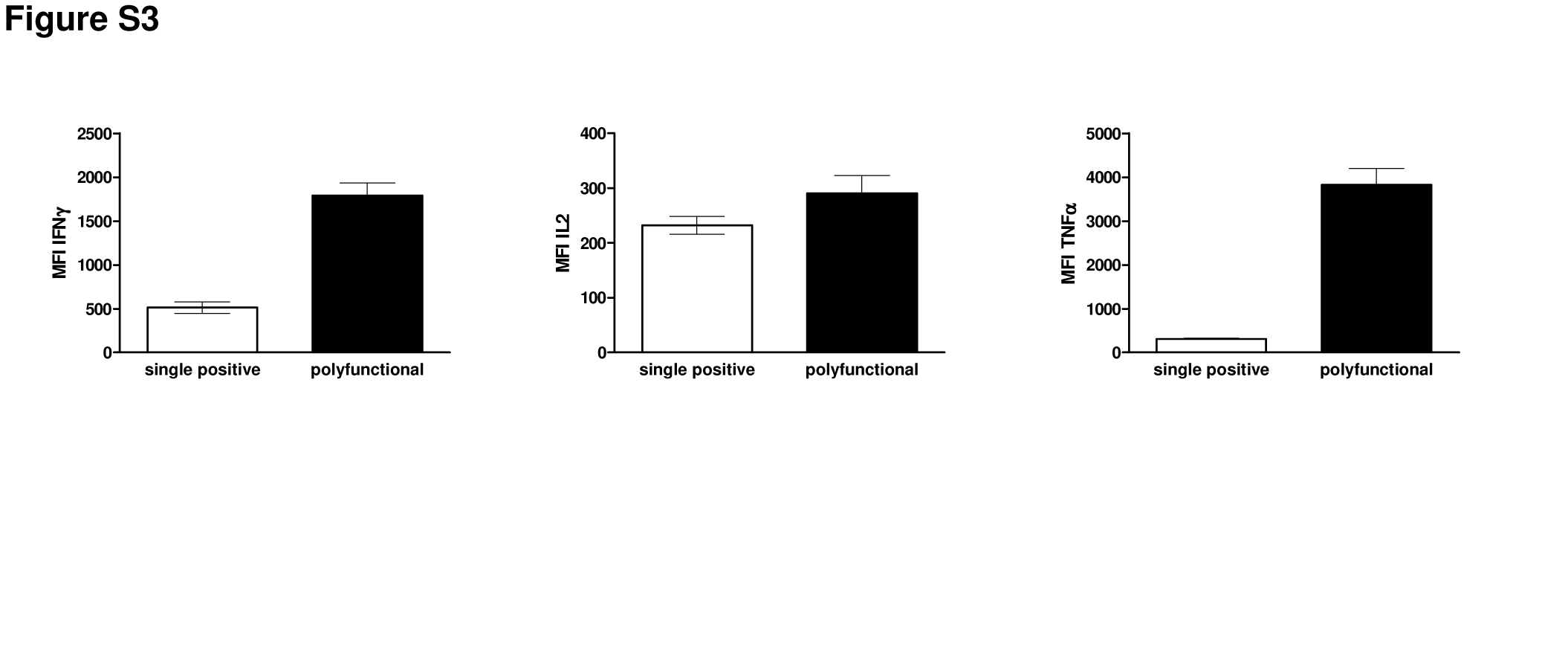

Supplement: Figure S3 — Polyfunctional CD4+ T cells produce higher cytokine levels on a per cell basis than single cytokine-producing CD4+ T cells. Two weeks after the last immunization with HIVBr18, spleen cells from 6 BALB/c mice were labeled with CFSE and cultured in the presence of pooled HIV-1 peptides or medium only for 4 days. On day 4, cells were pulsed for 12 hours with pooled HIV-1 peptides or medium in the presence of costimulatory antibody and Brefeldin. Multiparameter flow cytometry was used to identify polyfunctional and single cytokine-producing CD3+CD4+ T cells. Intracellular cytokine levels expressed as MFI values are compared for CFSElow cells producing all 3 tested cytokines (polyfunctional cells) and CFSElow cells producing a single cytokine. MFI values for IFNγ (left),TNFα (middle) and IL-2 (right). (TIF) [file pone.0016921.s003.tif]

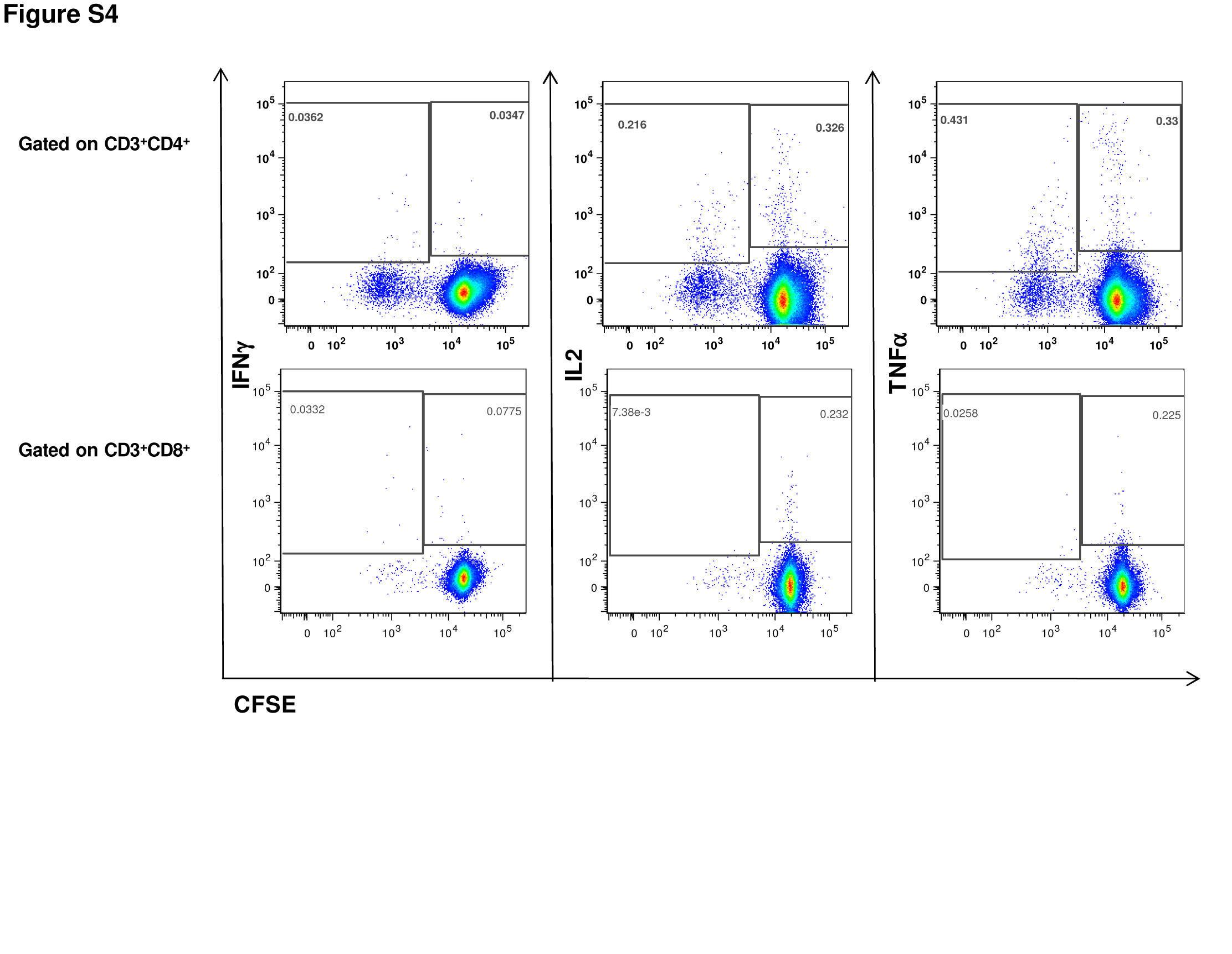

Supplement: Figure S4 — Proliferative responses and cytokine production in splenocytes from pVAX1 immunized mice. Two weeks after the last immunization with the empty vector pVAX1, spleen cells from 6 BALB/c mice were labeled with CFSE and cultured in the presence of pooled HIV-1 peptides or medium only for 4 days. On day 4, cells were pulsed for 12 hours with pooled HIV-1 peptides or medium in the presence of costimulatory antibody and Brefeldin A. CFSE and intracellular cytokine staining were used to simultaneously assess proliferation and IFNγ, TNFα or IL-2 production. Frequencies of antigen-specific cytokine-producing T cells in proliferating (CFSElow) and non proliferating (CFSEhi) gates are displayed. (TIF) [file pone.0016921.s004.tif]
