# Supplementary material for: A DNA Vaccine Encoding Multiple HIV CD4 Epitopes Elicits Vigorous Polyfunctional, Long-Lived CD4+ and CD8+ T Cell Responses
Source: PLoS One. 2011 Feb 11;6(2):e16921. doi: 10.1371/journal.pone.0016921 (PMC3037933; doi:10.1371/journal.pone.0016921)
Supplement: Table S1 — Peptide binding predictions for H-2d MHC class I and class II. (PDF) [file pone.0016921.s005.pdf]

Table S1

Peptide binding predictions for H-2d MHC class I and class II.

Peptide	Sequence	Prediction score by PREDBALB/c - binding at threshold= 8			
		MHC class II alleles		MHC class I alleles	
		I-Ad	I-Ed	H-2Kd	H-2Dd
**p17(73-89)**	EELRSLYNTVATLYCVH	**9.5**	**9.64**	**9.4**	**9.66**
**p24 (33-45)**	SPEVIPMFSALSE	**9.1**	4.96	6.1	**8.82**
**p24(131-150)**	KRWIILGLNKIVRMYSPTSI	**9.5**	**9.7**	**8.9**	**8.96**
**p6 (32-46)**	DKELYPLASLRSLFG	**9.1**	**8.82**	6.1	**9.4**
**pol (63-77)**	QRPLVTIKIGGQLKE	**10**	4.96	6.1	**9.44**
**pol (136-150)**	TPVNIIGRNLLTQIG	**8.3**	**8.14**	6.1	**9.6**
**pol (785-799)**	GKIILVAVHVASGYI	**9.7**	**9.76**	**8.98**	**9.2**
**gp41(261-276)**	RDLLLIVTRIVELLGR	**9.5**	**9.5**	7.78	**8.66**
**gp160 (19-31)**	TMLLGMLMICSAA	**9.6**	7.14	**8**	7.34
**gp160(174-185)**	ALFYKLDVVPID	**9.6**	**9.5**	7.7	**9.7**
**gp160 (188-201)**	NTSYRLISCNTSVI	**9.5**	**8.7**	**8.3**	7.3
**gp160 (481-498)**	SELYLYKVVKIEPLGVAP	**9.5**	**9.8**	**8.42**	**9.78**
**rev(11-27)**	ELLKTVRLIKFLYQSNP	**9.1**	**9.7**	7.8	**8.58**
**vpr(58-72)**	EAIIRILQQLLFIHF	**8.9**	**8.28**	7.74	**8.18**
**vpr (65-82)**	QQLLFIHFRIGCRHSRIG	**9.2**	**9.9**	7	**9.2**
**vif (144-158)**	SLQYLALVALVAPKK	**9.7**	**9.5**	**8.58**	**9.4**
**vpu(6-20)**	VLAIVALVVATIIAI	**9.6**	**9.4**	7.84	**8.96**
**nef (180-194)**	VLEWRFDSRLAFHHV	**9.7**	**9.5**	5.7	7.9

Scores from the PRED$^{BALB/c}$ algorithm range from 0 to 10; peptides with scores above 8 are predicted to bind and thus highlighted in bold.
